# The association between IGF‐1 plasma levels and Alzheimer's disease in the Alzheimer’s Disease Neuroimaging Initiative study

**DOI:** 10.1002/alz.093354

**Published:** 2025-01-09

**Authors:** Jermaine Ross, Yoonjeong Cha, Mohamedi Kagalwala

**Affiliations:** ^1^ Alleo Labs, San Francisco, CA USA

## Abstract

**Background:**

Insulin‐like Growth Factor‐1 (IGF‐1) and its receptor (IGF‐1R) are known to play a role in biological aging. Several studies have explored the correlation between serum levels of IGF‐1 and Alzheimer's‐related dementia (AD). However, conflicting reports exist regarding whether elevated or reduced IGF‐1 levels increase the risk of AD. The focus of this study is to investigate the relationship between IGF‐1 and AD participates in the Alzheimer’s Disease Neuroimaging Initiative (ADNI) study.

**Methods:**

We analyzed calculated readouts from biomarkers assessed in the study performed by Biomarkers Consortium Plasma Proteomics Project and made available in the ADNI online repository. The study analyzed plasma samples taken from ADNI participants at baseline and 12 months after enrollment. The samples were processed using a 190‐analyte multiplex immunoassay panel, which included a plasma readout for IGF‐1, measured in ng/mL for all 556 subjects. A Cohen's kappa value was calculated to determine an optimal cutoff for distinguishing AD cases compared to controls. A standard t‐test was performed to assess significance between groups.

**Result:**

At baseline, univariate analysis using a t‐test revealed a significant increase in IGF‐1 plasma levels in AD subjects compared to cognitively normal controls (p < 0.05). We also observed higher IGF‐1 levels in mild cognitive impairment (MCI) subjects compared to controls (p < 0.05). There was no significant difference between MCI and AD subjects. These findings are consistent with previous reports showing higher levels of IGF‐1 in animal models of AD, including transgenic mice expressing mutant human amyloid precursor protein and presenilin‐1. The optimal cutoff that maximizes Cohen's Kappa is >9.8 ng/mL of IGF‐1. In contrast, we did not observe any notable difference in IGF‐1 levels in either AD vs normal or MCI vs normal comparisons after 12 months, suggesting a potential temporal nature of the biomarker.

**Conclusions:**

In this study, we found a significant but transient relationship between IGF‐1 and Alzheimer’s‐related dementia. Given current focus on the development of blood‐based biomarkers, these results highlight a putative biomarker for stratifying Alzheimer’s risk, but additional work is required to understand the temporal aspect of IGF‐1 during disease progression.

